# Conservatism in the Treatment of Inflammation of Tubes and Ovaries

**Published:** 1901-10

**Authors:** 


### Conservatism in the Treatment of Inflammation of Tubes and Ovaries.

In the September number of the *St. Paul Medical Journal* Dr. J. L. Rothrock makes a plea for the conservative plan of treatment in inflammatory conditions of the tubes and ovaries. He has examined microscopically a great number of diseased tubes and ovaries and has found that many specimens which have the naked eye appearance of being almost completely disorganized are really very little changed in structure. He calls attention to the fact that even neglected pus tubes rarely ever cause death since as the result of repeated attacks of localized peritonitis the pus is usually well walled off, the inflammation in time dying out and leaving a harmless accumulation of sterile pus.

Many attacks of pelvic inflammation never reach the stage of pus formation. According to Zweifel only about four per cent. terminate in suppuration.

The common belief that tubal and ovarian infections travel along the mucous membrane of the tubes from the uterus is erroneous. Except in the case of the gonococcus all such infections occur, as in other localities, through the lymphatics and blood-vessels.

The results of laparotomy for the removal of tubes and ovaries in cases of gonococcus infection are disappointing. Frith says that "fully thirty per cent. of the patients upon whom laparotomy has been performed for the relief of pyosalpinx were not in the least benefited, and that it is the most unsatisfactory operation that the modern gynecologist performs. Though the patient is often for a time apparently benefited the symptoms return. In addition, she suffers other distressing symptoms, resulting from the loss of her ovaries."

In the treatment of patients suffering from tubo-ovarian disease the author resorts to local treatment directed chiefly to the uterine cavity. All treatment is of course conducted under strict antiseptic precautions. He begins with irrigation of the uterine cavity with 1-5000 bichloride mercury solution, followed by fifty per cent. ichthyol in glycerine. If the os be stenosed the cervix must be dilated, preferably by sterile sea tangle tents. In the acute stage the patient must be put to bed and cold applications used to the abdomen. For local sensitiveness the application of tincture of iodine to the vaginal vault followed by wool tampon impregnated with fifty per cent. ichthyol in glycerine gives good results.

